# Supplementary material for: Differences between Kidney Transplant Recipients from Deceased Donors with Diabetes Mellitus as Identified by Machine Learning Consensus Clustering
Source: J Pers Med. 2023 Jul 3;13(7):1094. doi: 10.3390/jpm13071094 (PMC10381319; doi:10.3390/jpm13071094)
Supplement: Supplementary file 1 [file jpm-13-01094-s001.zip › jpm-2435381-supplementary.pdf]

Supplementary Methods

Cluster derivation

We applied an unsupervised ML approach to develop clinical phenotypes of recipients of diabetic deceased donor kidney transplants in the UNOS/OPTN database by conducting unsupervised consensus clustering.[1] We performed consensus clustering analysis on the whole study population. We initially assessed the distribution and missingness in phenotyping variables. Subsequently, missing data were imputed through multiple imputation using multivariate imputation by chained equations (MICE),[2] and non-normal data were z-score normalized. Multiple imputation is a widely used approach to estimate variables when data are missing at random. MICE is optimal when less than 30% of a variable's data are missing.[3-8] All of the extracted variables in our study had missing data ≤5% (Table S1). We subsequently applied clustering using the consensus cluster algorithm. The algorithm begins by subsampling a proportion of items and a proportion of features from a data matrix. Each subsample is then partitioned into up to groups (k) by a user-specified clustering algorithm. This process is repeated for a specified number of times. Pairwise consensus values, defined as 'the proportion of clustering runs in which two items are grouped together', are calculated and stored in a consensus matrix (CM) for each cluster. Clustering settings used were as follows: maximum number of clusters, 10; number of iterations, 100; subsampling fraction, 0.8; clustering algorithm, K-means; Euclidean distance).[1] The number of potential clusters ranges from 2 to 10, to avoid producing an excessive number of clusters that would not be clinical useful. Pairwise consensus values, defined as 'the proportion of clustering runs in which two items are [grouped] together[1], are calculated and stored in a CM for each k. Then for each k, a final agglomerative hierarchical consensus clustering using distance of 1−consensus values is completed and pruned to k groups, which are called consensus clusters.

The clustering algorithm is to maximize the potential number of clusters while maintaining high cluster consensus. The optimal number of clusters was determined by examining the CM heat map, cumulative distribution function, cluster-consensus plots with the within-cluster consensus scores, and the proportion of ambiguously clustered pairs (PAC).[9, 10] The within-cluster consensus score, ranging between 0 and 1, is defined as the average consensus value for all pairs of individuals belonging to the same cluster.[10] A value closer to one indicates better cluster stability.[10] PAC, ranging between 0 and 1, is calculated as the proportion of all sample pairs with consensus values falling within the predetermined boundaries.[9] A value closer to zero indicates better cluster stability.[9] To examine the cluster profile, we calculated and graphically displayed the standardized mean differences of the variables between each cluster and the overall study population. Calculation of the standardized difference of each parameter used the cutoff of ±0.3 to show subgroup features with the key features for each cluster.

All cluster derivation analyses were performed using R, version 4.0.3 (RStudio, Inc., Boston, MA; http://www.rstudio.com/), with the packages of ConsensusClusterPlus (version 1.46.0)[10]. We imputed missing data through multivariable imputation by chained equation (MICE) method.[2] All analyses were two-tailed, and P value < .05 was considered statistically significant.

Supplementary Figure 1. Consensus matrix heat map (k = 2) depicting consensus values on a white to blue color scale of each cluster

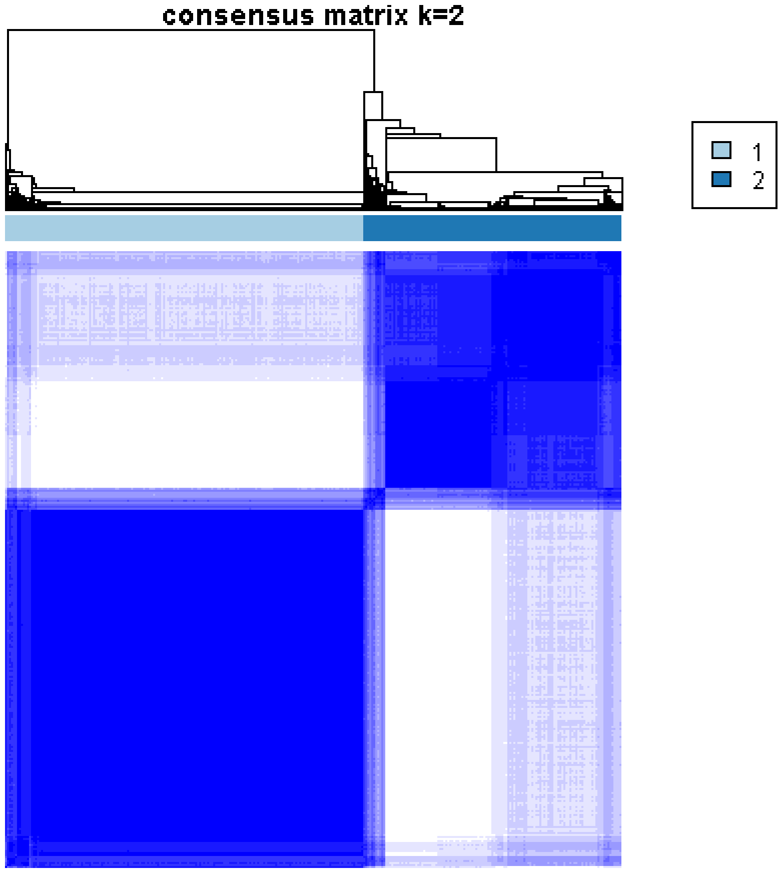

Supplementary Figure 2. Consensus matrix heat map (k = 3) depicting consensus values on a white to blue color scale of each cluster

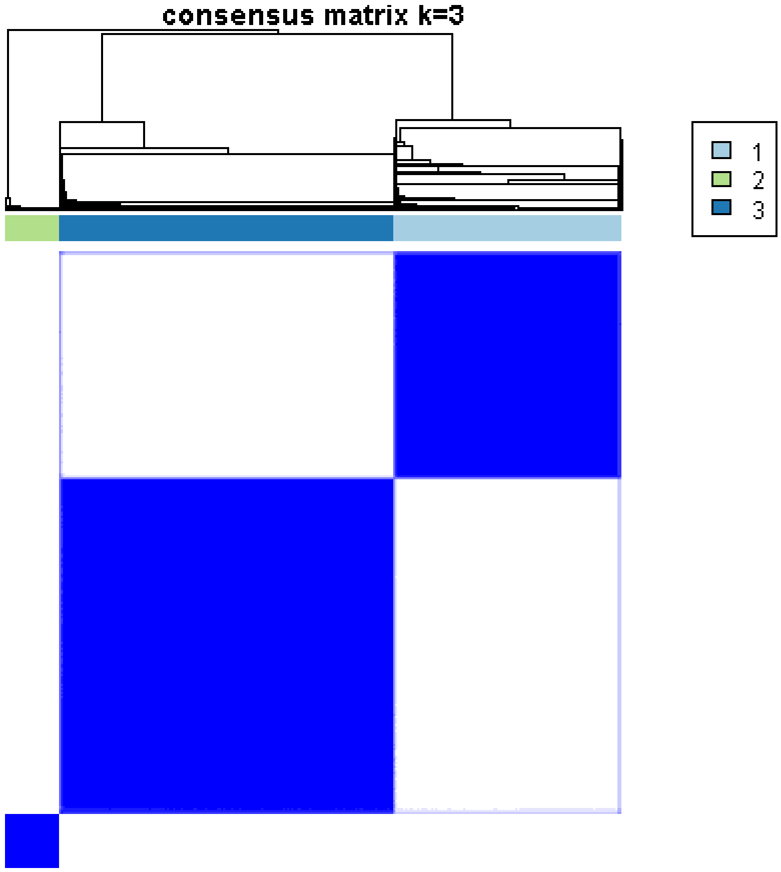

Supplementary Figure 3. Consensus matrix heat map (k = 4) depicting consensus values on a white to blue color scale of each cluster

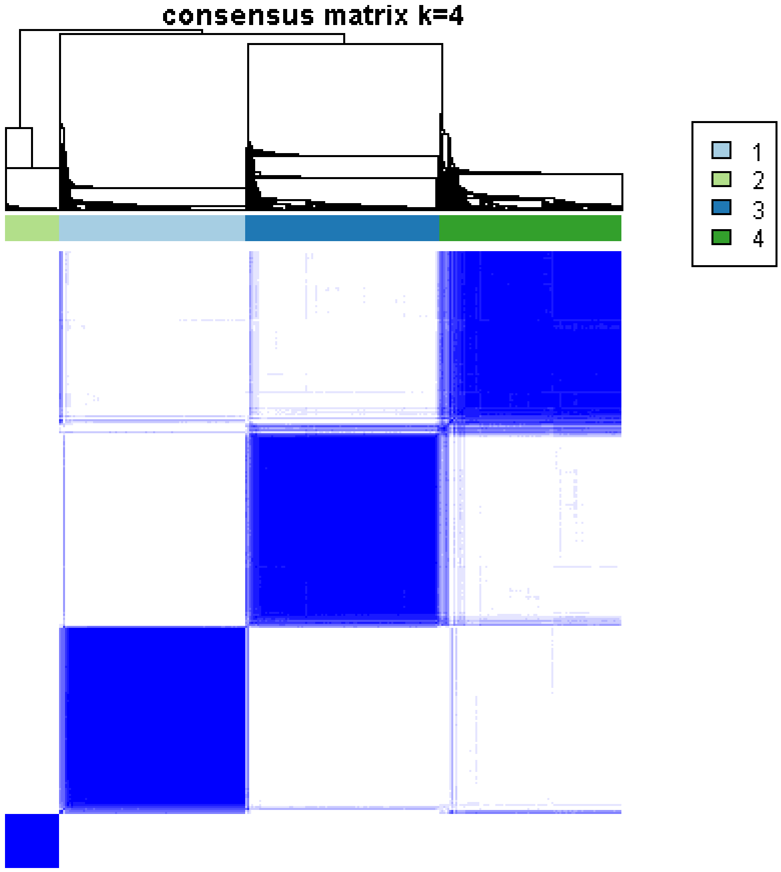

Supplementary Figure 4. Consensus matrix heat map (k = 5) depicting consensus values on a white to blue color scale of each cluster

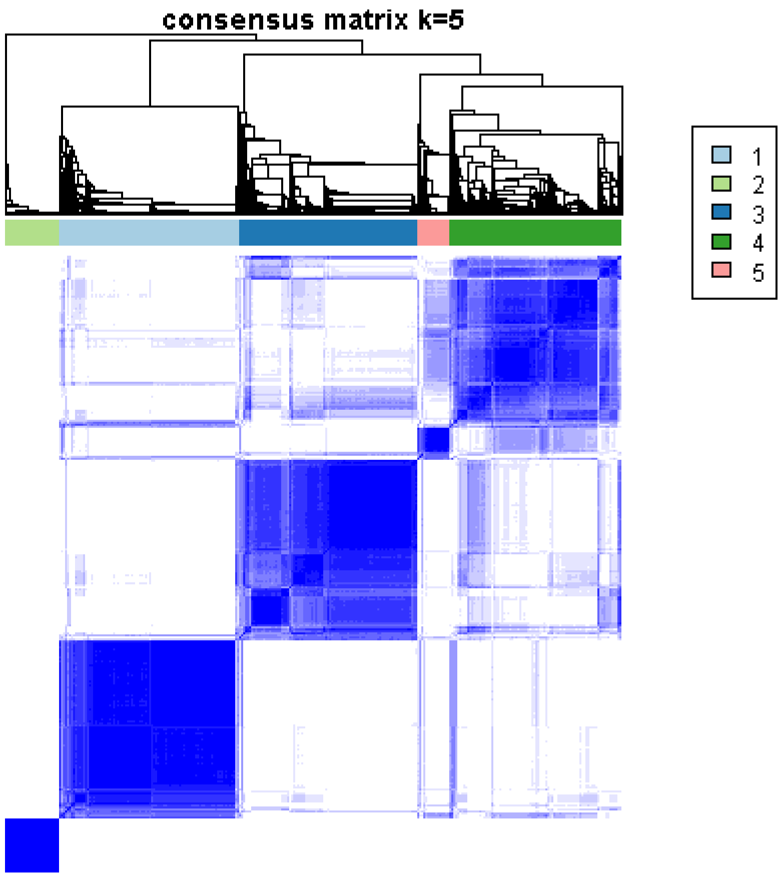

Supplementary Figure 5. Consensus matrix heat map (k = 6) depicting consensus values on a white to blue color scale of each cluster

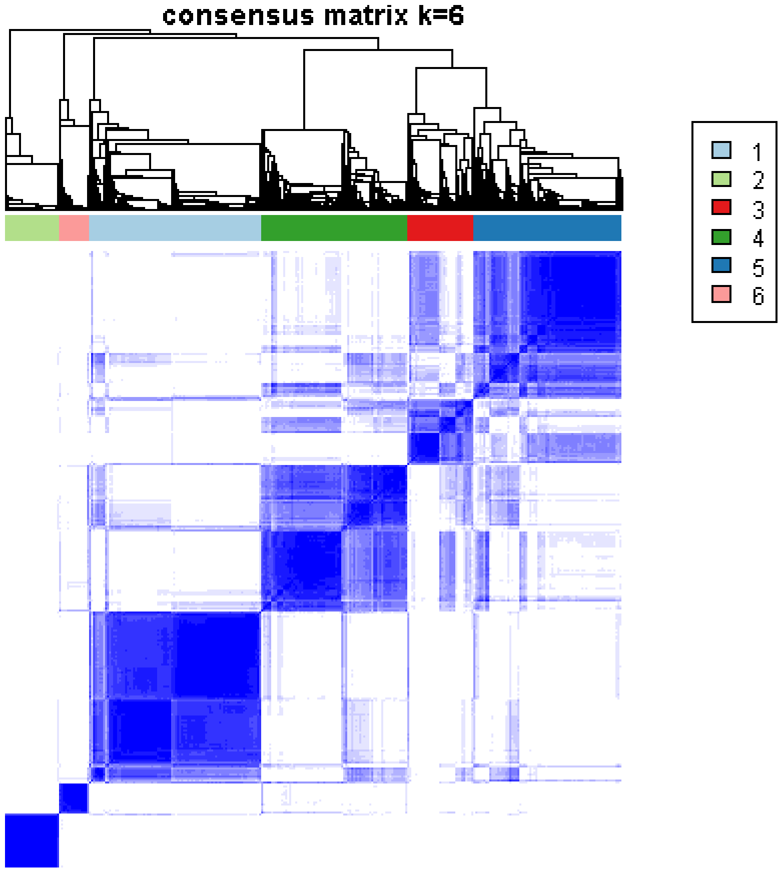

Supplementary Figure 6. Consensus matrix heat map (k = 7) depicting consensus values on a white to blue color scale of each cluster

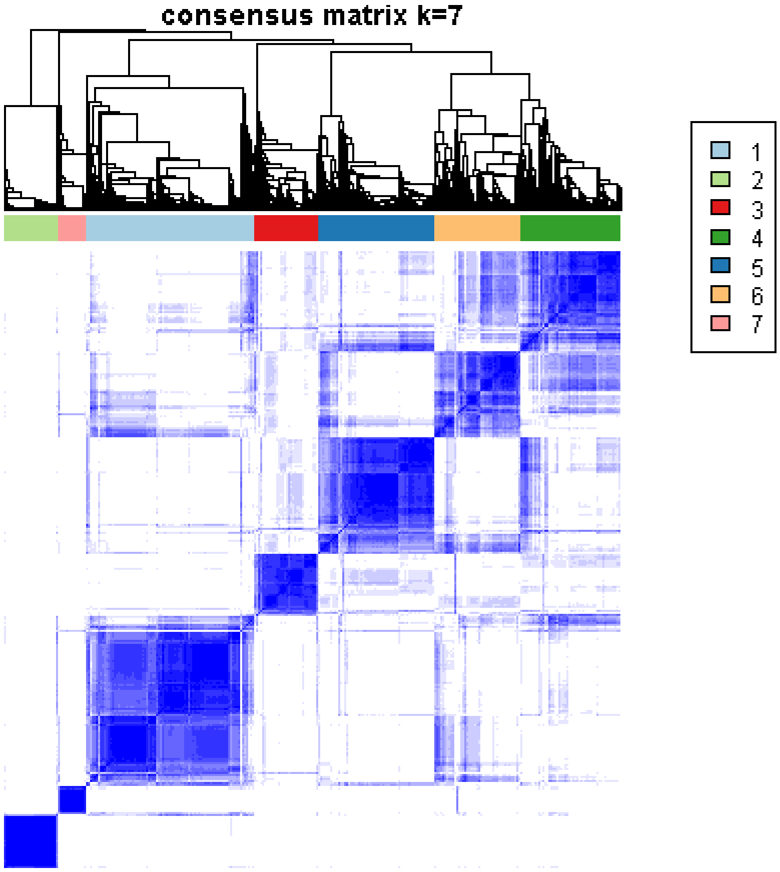

Supplementary Figure 7. Consensus matrix heat map (k = 8) depicting consensus values on a white to blue color scale of each cluster

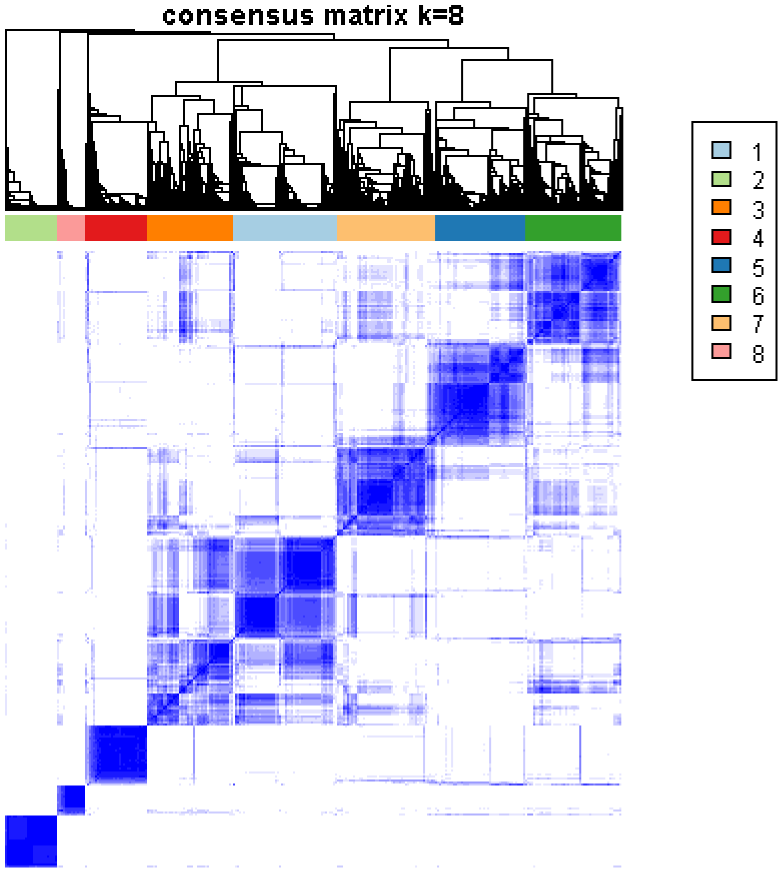

Supplementary Figure 8. Consensus matrix heat map (k = 9) depicting consensus values on a white to blue color scale of each cluster

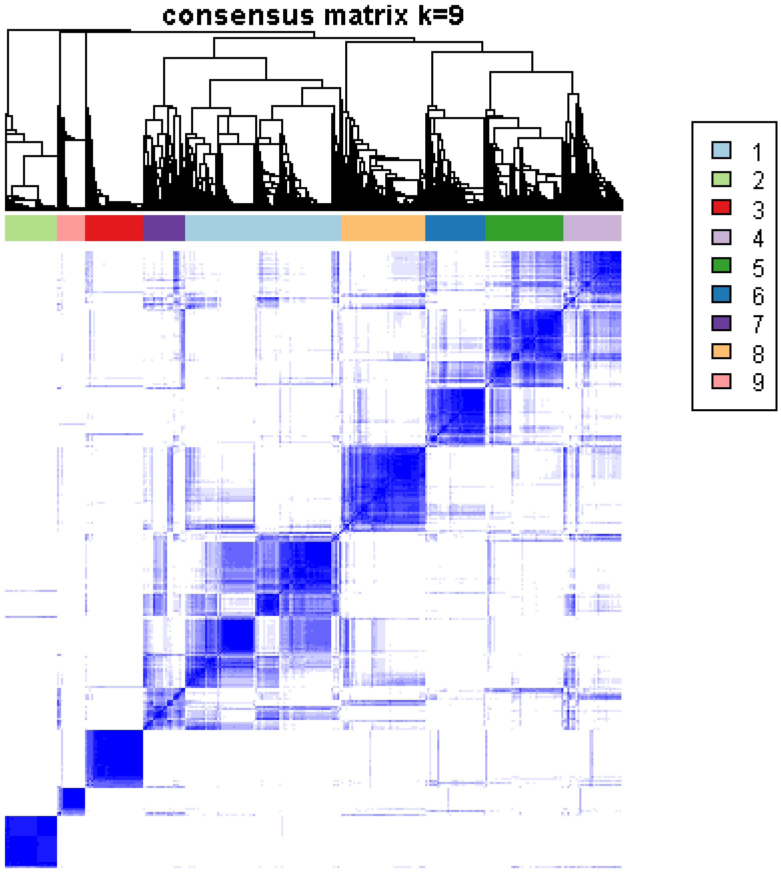

Supplementary Figure 9. Consensus matrix heat map (k = 10) depicting consensus values on a white to blue color scale of each cluster

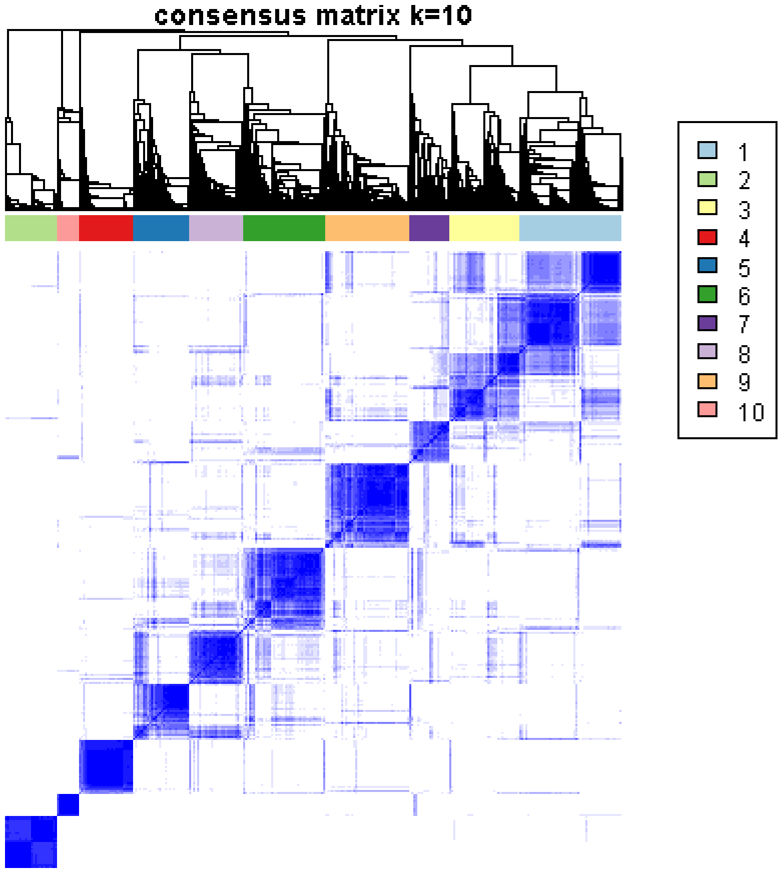

References

1 Monti S, Tamayo P, Mesirov J, Golub T. Consensus clustering: a resampling-based method for class discovery and visualization of gene expression microarray data. Machine learning. 2003: 91

2 Van Buuren S, Groothuis-Oudshoorn K. mice: Multivariate imputation by chained equations in R. Journal of statistical software. 2011: 1

3 Jannat-Khah DP, Unterbrink M, McNairy M, Pierre S, Fitzgerald DW, Pape J, Evans A. Treating loss-to-follow-up as a missing data problem: a case study using a longitudinal cohort of HIV-infected patients in Haiti. BMC Public Health. 2018: 1269 [PMID: 30453995 10.1186/s12889-018-6115-0: 10.1186/s12889-018-6115-0]

4 Knol MJ, Janssen KJ, Donders AR, Egberts AC, Heerdink ER, Grobbee DE, Moons KG, Geerlings MI. Unpredictable bias when using the missing indicator method or complete case analysis for missing confounder values: an empirical example. J Clin Epidemiol. 2010: 728 [PMID: 20346625 10.1016/j.jclinepi.2009.08.028: 10.1016/j.jclinepi.2009.08.028]

5 White IR, Carlin JB. Bias and efficiency of multiple imputation compared with complete-case analysis for missing covariate values. Stat Med. 2010: 2920 [PMID: 20842622 10.1002/sim.3944: 10.1002/sim.3944]

6 White IR, Royston P, Wood AM. Multiple imputation using chained equations: Issues and guidance for practice. Stat Med. 2011: 377 [PMID: 21225900 10.1002/sim.4067: 10.1002/sim.4067]

7 Hedden SL, Woolson RF, Carter RE, Palesch Y, Upadhyaya HP, Malcolm RJ. The impact of loss to follow-up on hypothesis tests of the treatment effect for several statistical methods in substance abuse clinical trials. J Subst Abuse Treat. 2009: 54 [PMID: 19008067 10.1016/j.jsat.2008.09.011: 10.1016/j.jsat.2008.09.011]

8 Donders ART, Van Der Heijden GJ, Stijnen T, Moons KG. A gentle introduction to imputation of missing values. Journal of clinical epidemiology. 2006: 1087

9 Șenbabaoğlu Y, Michailidis G, Li JZ. Critical limitations of consensus clustering in class discovery. Sci Rep. 2014: 6207 [PMID: 25158761 10.1038/srep06207: 10.1038/srep06207]

10 Wilkerson MD, Hayes DN. ConsensusClusterPlus: a class discovery tool with confidence assessments and item tracking. Bioinformatics. 2010: 1572
